# Factors associated with severe adverse events following COVID-19 vaccine: a multicenter study in the Casablanca-Settat region of Morocco

**DOI:** 10.11604/pamj.2025.52.63.48603

**Published:** 2025-10-07

**Authors:** Rachid Nouaji, Fatima Zahra Meski, Nadia Hermani, Rachid Razine, Majdouline Obtel, Nada Bennani Mechita

**Affiliations:** 1 Regional Directorate of the Ministry of Health and Social Protection, Casablanca Settat, Morocco; 2 National School of Public Health, Rabat, Morocco; 3 Laboratory of Biostatistics, Clinical Research and Epidemiology, Laboratory of Community Health, Preventive Medicine and Hygiene, Department of Public Health, Faculty of Medicine and Pharmacy, University Mohammed V, Rabat, Morocco; 4 Pedagogy and Research Unit of Public Health, Department of Public Health, Faculty of Medicine and Pharmacy, University Mohammed V, Rabat, Morocco

**Keywords:** COVID-19, COVID-19 vaccines, vaccination/adverse effects, immunization, pharmacovigilance

## Abstract

**Introduction:**

in January 2021, a national COVID-19 vaccine campaign was launched in Morocco. The national pharmacovigilance system was strengthened to assess any adverse events related to the COVID-19 vaccine. The objective of this study was to analyse the factors associated with severe Adverse Events Following Immunization (AEFI) related to COVID-19 vaccines in the Casablanca-Settat region in Morocco.

**Methods:**

this multicentric, cross-sectional study was conducted among individuals who reported AEFI after COVID-19 vaccines. Factors associated with the occurrence of severe AEFI were analysed using logistic regression.

**Results:**

a total of 1257 reports (2538 AEFI) were analysed. The AstraZeneca vaccine was responsible for 65% of the AEFI. 74% of cases occurred after the first dose, and 92% were considered non-serious. Among the 95 serious cases, 51% presented their first AEFI within 24 hours of vaccination. Age ≥65 years (OR = 2.63; p < 0.001), male gender (OR = 1.75; p = 0.03), and a number of AEFI per patient ≥3 (OR = 2.08; p < 0.001) were associated with serious AEFI.

**Conclusion:**

although the majority of cases were non-serious, this study highlights the importance of vigilance, particularly for at-risk populations such as the elderly, men, and patients with a high number of AEFI.

## Introduction

Since its emergence in December 2019, the SARS-CoV-2 virus has rapidly spread across the globe, infecting millions and causing a significant number of deaths. In response to this global health crisis, governments, health organisations, and researchers collaborated to find solutions and implement strategies to contain the spread of the virus. Vaccination, in particular, has been essential in preventing the overload of healthcare systems and mitigating its consequences [1]. Emergency use authorisation was granted to vaccines before the completion of conventional phases of clinical trials, raising concerns about their safety. Numerous studies and analyses have been conducted worldwide to evaluate the efficacy and safety of COVID-19 vaccines [2].

On March 2^nd^, 2020, Morocco confirmed its first case of COVID-19. However, as early as January 2020, the Moroccan government had already established a strategic plan to combat the virus's spread. This plan included measures such as wearing masks, social distancing, handwashing, ventilation, and periods of lockdown. Despite these efforts, the ongoing spread of the virus pushed Morocco to intensify its strategies, placing vaccination at the center of its fight. Thus, starting in January 2021, a national vaccination campaign was launched, utilising Sinopharm, AstraZeneca, Pfizer-BioNTech, and Johnson and Johnson vaccines. The campaign initially targeted at-risk groups, including healthcare professionals, authority personnel, the elderly, and those with comorbidities or chronic illnesses. It then gradually expanded to include the entire population, including youths aged 12 to 17 [3].

In the context of the massive use of vaccines developed in record time using new technologies, the national pharmacovigilance system was adapted and strengthened to quickly identify and assess any adverse events related to COVID-19 vaccination. This was achieved through improved communication between the different levels (national, regional, and local) of monitoring Adverse Event Following Immunization (AEFI). Simultaneously, training programs were launched for regional coordinators and local pharmacovigilance points. Additionally, revisions of data collection and analysis protocols were made, accompanied by extensive awareness campaigns for healthcare professionals and the general public regarding the importance of reporting AEFIs.

The Casablanca-Settat Region (CSR), one of the twelve regions of the Kingdom of Morocco, played a vital role in implementing the national vaccination campaign. As part of the monitoring of AEFI related to COVID-19, and in collaboration with the National Pharmacovigilance Center (NPC), the Regional Pharmacovigilance Unit (RPU) was established to comprehensively manage notifications from various provinces and prefectures of the region. Through the centralisation of this data, the RPU was able to create a regional database on AEFIs. This database provides a valuable opportunity to conduct an in-depth study of reported events, allowing for a better understanding of the safety profile of the COVID-19 vaccines used in the CSR. This study aimed to explore the nature, frequency, and factors associated with severe adverse events following immunisation (AEFI) against COVID-19 recorded in the CSR between January and November 2021.

## Methods

**Study type and population:** this multicenter, cross-sectional study focuses on individuals aged 12 and over who reported adverse events after being vaccinated against COVID-19 in the CSR, Morocco, between January 29^th^ and November 23^rd^, 2021.

### Study population

***Inclusion criteria:*** participants included in this study had to meet the following criteria: aged 12 years or older; have experienced one or more Adverse Events Following Immunization (AEFI) against COVID-19 between January 29^th^ and November 23^rd^, 2021, in provinces and prefectures of the Casablanca-Settat region and have received a dose of one of the following COVID-19 vaccines: AstraZeneca, Sinopharm, Pfizer, or Janssen.

***Exclusion criteria:*** participants who had received any other vaccinations during the study period

**Data collection:** the data were extracted from the regional Adverse Events Following Immunization (AEFI) notification database managed by the Regional Pharmacovigilance Unit (RPU). This collection covered the period from January 29, 2021, to November 23, 2021. The AEFI notification database related to COVID-19 vaccination was compiled from AEFI notification forms. These forms contained information on the patient, the AEFIs, the administered vaccine, and the AEFI reporter. Patients reported AEFI during consultations at the time of vaccination for immediate AEFIs or during medical consultations in Ministry of Health facilities for delayed AEFIs. Once the forms were completed, they were sent to provincial or prefectural pharmacovigilance focal points for verification and finalisation. They were then forwarded to the RPU by mail, email, or telephone for severe AEFIs. The data were subsequently verified, supplemented, and entered into an Excel file, forming the regional AEFI notification database. This study was based on a secondary analysis of the national AEFI notification database related to COVID-19 vaccination. AEFI were categorised according to the Medical Dictionary for Regulatory Activities classification (MedDRA) [4]. An adverse event was classified as serious if it resulted in: i) death, ii) hospitalisation or prolongation of hospitalisation, iii) life-threatening, iv) sequelae or disability, v) congenital anomaly, vi) medical intervention to prevent permanent impairment or disorder [5].

**Data analysis:** data analysis was conducted using JAMOVI software (version 2.4.14). Descriptive statistics summarised sociodemographic and clinical characteristics: means with standard deviations (SD) for normally distributed continuous variables, and medians with interquartile ranges (IQR) for non-normally distributed variables. Categorical variables were presented as frequencies and percentages. Associations between the severity of adverse events and explanatory variables were assessed using the Chi-square test or Fisher's exact test, as appropriate. Multivariable logistic regression was performed to identify independent factors associated with severe AEFI, with results expressed as odds ratios (OR) and 95% confidence intervals (CI). A p-value < 0.05 was considered statistically significant.

**Ethical approval:** patient anonymity has been strictly preserved. Data collection was authorised by the Regional Director of the Ministry of Health for the Casablanca-Settat Region (CSR) and the study was approved by the Ethics Committee of Biomedical Research of the Faculty of Medicine and Pharmacy of Rabat (No: B-24).

## Results

**Figure 1:**
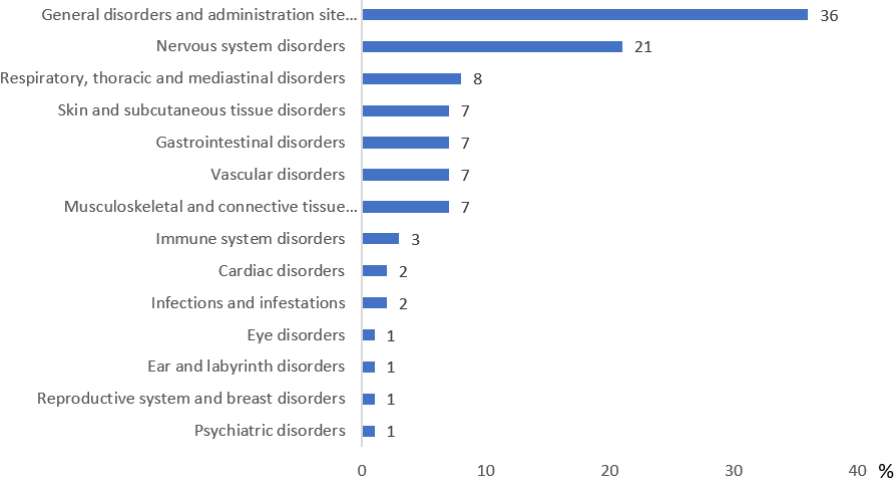
distribution of COVID-19 AEFI by MedDRA SOC categories in the population of the Casablanca-Settat region, (January - November, 2021)

**Demographic and clinical characteristics of the vaccinated population:** during the study period, 1,257 cases of AEFI were reported, corresponding to a total of 2,538 individual events. This represented an overall incidence of 2.6 AEFI per 10,000 vaccine doses, based on the 9,754,275 doses administered. The sociodemographic and clinical characteristics of cases are summarised in Table 1. Of all cases, 787 (63%) were female, with a median age of 58 years [IQR: 45-67]. The 18-64 years age group was the most represented (61%). A history of at least one chronic medical condition was reported in 478 cases (38%), most commonly hypertension (HTN) and diabetes mellitus. The AstraZeneca vaccine was associated with the largest number of reports (800 cases, 65%). The majority of AEFI occurred after the first dose (74.4%). Most cases were classified as non-serious (1,161; 92%), and 70% developed symptoms within 24 hours of vaccination. Among the 2,538 reported events, the most frequent category was general disorders and administration site conditions (906 events, 36%), followed by nervous system disorders (528 events, 21%). The distribution of AEFI by system organ class is detailed in Figure 1

**Table 1:** socio-demographic and clinical characteristics of the population vaccinated against COVID-19 in the Casablanca-Settat region (January - November 2021)

Characteristics	n (%)
**Gender (n =1249) b**	
Female	787 (63)
Male	462 (37)
**Age (years) (n =847) b**	58 [45; 67] a
12-17	51 (06)
18-64	520 (61)
≥ 65	276 (33)
**Medical history (n =1257) b**	
Yes	482 (38)
Hypertension	166 (34)
Diabetes	114 (24)
Heart diseases	48 (10)
Immune system disorders	46 (10)
Other disorders	108 (22)
**Vaccine (n =1224) b**	
AstraZenica	800 (65)
Sinopharm	337 (27)
Pfizer	81 (07)
Janssen	06 (01)
**Vaccine dose (n =1123) b**	
First dose	835 (74)
Second dose	276 (25)
Third dose	12 (01)
**Severity (n =1257) b**	
Yes	95 (08)
No	1161 (92)
**Time to onset of the first AEFI (n =1132) b**	
≤ 1H	355 (31)
≤ 24	445 (39)
≤ 07 days	264 (23)
> 07 days	74 (07)

a: Expressed as median [Q25 - Q75]; b: Expressed as n (%); AEFI: Adverse events following immunization

**Figure 2:**
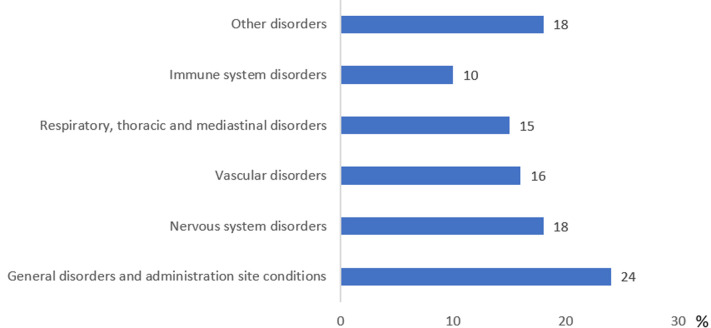
distribution of COVID-19 AEFI in severe cases vaccinated in the Casablanca-Settat region, (January - November 2021)

**Demographic and clinical characteristics of cases presenting with severe AEFI:** the RPU recorded 95 notifications of AEFI classified as severe. The average age of patients was 62 ± 16 years, with a sex ratio close to 1 (F/M ≈1). More than half of the cases (53%) were 65 years or older. Additionally, 54% had at least one medical history, with the most common conditions being hypertension (27%), diabetes (21%), and heart disease (14%). Seventy-six per cent of severe cases were reported following the administration of the AstraZeneca vaccine, with the majority (71%) occurring after the first dose. Furthermore, 50% of severe cases exhibited their initial AEFI within 24 hours post-vaccination. Regarding the health outcomes of severe cases, 54% achieved recovery. Following the classification of AEFI in severe cases according to the Medical Dictionary for Regulatory Activities System Organ Class system (MedDRA SOC), a total of 215 AEFIs were identified. Of these, general disorders and administration site reactions were the most frequently reported, representing 24% of total AEFI. Nervous system disorders were also commonly reported (18%). Other findings are presented in Figure 2.

Factors associated with the severity of COVID-19 AEFI in the Casablanca-Settat region, Morocco (January - November 2021); N = 95: the bivariate analysis (Table 2) showed significant associations between AEFI severity and several factors: age (p < 0.001), gender (p = 0.008), time to onset of the first AEFI (p = 0.001), number of AEFI per patient (p = 0.03), and history of heart disease (p = 0.021). In univariate logistic regression (Table 3), the following were significantly associated with severe AEFI: age ≥65 years (OR = 2.86; 95% CI: 1.74-4.68; p < 0.001), male gender (OR = 1.75; 95% CI: 1.15-2.67; p = 0.01), time to onset > 7 days (OR = 2.77; 95% CI: 1.46-5.27; p < 0.001), ≥3 AEFI per patient (OR = 1.60; 95% CI: 1.05-2.47; p = 0.03), and heart disease (OR = 2.56; 95% CI: 1.26-5.23; p = 0.01). After multivariable adjustment for age, gender, number of AEFI per patient, and heart disease, independent predictors of severe AEFI remained: age ≥65 years (OR = 2.63; 95% CI: 1.57-4.39; p < 0.001), male gender (OR = 1.75; 95% CI: 1.05-2.90; p = 0.03), and ≥3 AEFI per patient (OR = 2.08; 95% CI: 1.25-3.44; p < 0.001) (Table 3)

**Table 2:** comparison of characteristics and clinical presentation in severe vs non-severe AEFI after COVID-19 vaccination in the Casablanca-Settat region, Morocco

Variables	Severe case		χ2-p value
	Yes	No	
	N=95	N=1162	
**Age (years)**			<0.001
<65	33 (05.6)	558 (94.4)	
≥65	37 (14.5)	219 (85.5)	
**Gender**			0.008
Male	47 (10.2)	415 (89.8)	
Female	48 (06.1)	739 (93.9)	
**Medical history**			0.083
Yes	51 (06.5)	728 (93.5)	
No	44 (09.2)	434 (90.8)	
**Comorbidity:**			
**- Diabetes**			0.187
Yes	15 (10.3)	131 (89.7)	
No	80 (07.2)	1031 (92.8)	
**- Hypertension**			0.554
Yes	20 (08.5)	216 (91.5)	
No	75 (07.3)	946 (92.7)	
**- Hypertension -diabetes**			0.909
Yes	6 (07.9)	70 (92.1)	
No	89 (07.5)	1092 (92.5)	
**- Heart disease**			0.021
Yes	10 (16.4)	51 (83.6)	
No	85 (07.1)	1111 (92.9)	
**- Immune disorders**			0.875
Yes	4 (07.0)	53 (93.0)	
No	91 (07.6)	1109 (92.4)	
**Vaccine type**			0.093
Sinopharm	19 (05.6)	318 (94.4)	
AstraZeneca	71 (08.9)	729 (91.1)	
Pfizer and Janssen	4 (4.6)	83 (95.4)	
**Vaccine dose**			0.449
First	61 (07.3)	774 (92.7)	
Second and third	25 (08.7)	263 (91.3)	
**Onset of 1st AEFI**			0.001
≤ 07 days	76 (07.1)	988 (92.9)	
> 07 days	13 (17.6)	61 (82.4)	
**Number of AEFI/patient**			0.030
One and two	57 (06.5)	821(93.5)	
Three and more	38 (10.0)	341(90.00)	

Values are expressed as number (%); χ^2^: chi-square test

**Table 3:** univariate and multivariate logistic regression analyses of factors associated with severe adverse events following COVID-19 immunisation in the Casablanca-Settat region, 2021

Variables	Univariate analysis	p	Multivariate analysis	p
	OR (95% CI)		OR (95% CI)	
**Age (years) (ref: <65)**				
≥ 65	2.86 (1.74-4.68)	<0.01	2.63 (1.57-4.39)	<0.01
**Gender (ref: Female)**				
Male	1.74 (1.15-2.65)	0.01	1.75 (1.05-2.90)	0.03
**Medical history (ref: No)**				
Yes	1.45 (0.95-2.20)	0.09		
**Diabetes (ref: No)**				
Yes	1,48 (0,83-2,64)	0,19		
**Hypertension (ref: No)**				
Yes	1.17 (0.70-1.95)	0.56		
**Hypertension-diabetes (ref: No)**				
Yes	1.05 (0.44-2.49)	0.91		
**Heart disease (ref: No)**				
Yes	2.56 (1.26-5.23)	0.01	2.01 (0.86-4.66)	0.10
**Immune disorders (ref: No)**				
Yes	0.92 (0.33-2.60)	0.88		
**Vaccine type (ref: Sinopharm)**				
AstraZeneca	1.63 (0.97-2.75)	0.07		
Pfizer Janssen	0.81 (0.27-2.43)	0.70		
**Vaccine dose (ref: First)**				
Second and third	1.21 (0.74-1.96)	0.45		
**Onset of 1^st^ AEFI (ref: ≤ 07 days)**				
> 07 days	2.77 (1.46-5.27)	<0.01		
**Number of AEFI/patient (ref:1 and 2)**				
Three and more	1.61 (1.05-2.47)	0.03	2.08 (1.25-3.44)	<0,01

OR: Odds Ratio; CI: Confidence Interval; ref: reference group

## Discussion

We analysed 1257 AEFI notifications, totalling 2538 reported AEFI following the administration of 9,754,275 doses of COVID-19 vaccines, which translates to an overall rate of 2.6 AEFI per 10000 doses administered. This allowed us to investigate the nature, frequency, and factors associated with severe AEFI related to COVID-19 recorded in the CSR from January to November 2021. The national rate, however, was 7.3 per 10,000 doses. [5], This discrepancy could be explained by the database's exclusion of CSR notifications that were sent straight to the NPC. Furthermore, we lacked access to data from the "Yakadaliquah" platform, so a sizable portion of notifications were left out. In this study, the median age was 58 years [45 - 67], a result close to that of another study conducted in Morocco [6]. AEFI notifications concerned 63% of females, which is consistent with other studies conducted in Morocco [6,7]. However, a report from the NPC in Morocco showed a balanced sex ratio (≈1) [5]. The most common comorbidities found in this study, both in the total number of cases examined and in the severe cases, were heart disease, diabetes, and hypertension. Other research carried out in Morocco [6,7] and South Korea [8] has shown the same outcomes.

The AstraZeneca vaccine accounted for 65% of AEFI notifications among all reported cases and 76% of reports regarding severe cases. These findings are consistent with those reported by the National Pharmacovigilance Center (NPC) of Morocco and are supported by other studies conducted in Morocco [6,7], Algeria [9], Syria [10], and Germany [11]. In this study, the first dose was responsible for the majority of AEFI reports (74%), which is similar to several other results from Morocco [7], Ethiopia [12], and Iran [13]. This may be explained by the fact that the initial immune response is triggered by the first dosage of the vaccine, which in certain people can result in more severe reactions. Similar to the study from the CHU of Marrakech in Morocco, the distribution of reports based on the time to the beginning of the first AEFI revealed that the majority of AEFI (70%) happened within a relatively short time frame (≤24 hours) following vaccination. [7] and Ethiopia [12].

In this study, general disorders and administration site conditions represented the most reported category, with 914 (36%). Nervous system disorders were also frequently reported, with 533 (21%). These results are similar to those from the NPC, the CHU Marrakech in Morocco [7], and also those from Occitanie-East in France [14]. This study revealed that 95 cases (8%) presented at least one serious AEFI. Data from the United States Vaccine Adverse Event Reporting System (VAERS) indicated that approximately 1.52% of reported adverse events were classified as life-threatening [15]. These variations underscore the heterogeneity in reported AEFI severity across different populations and surveillance systems. One plausible explanation for this variability is the influence of racial and ethnic differences on susceptibility to adverse drug reactions [16]. The multivariate logistic regression model identified age, male gender, and the number of AEFIs per patient ≥ three as associated factors with severe adverse events. We observed that patients aged 65 years and older had an increased risk of developing severe AEFI compared to those under 65 (OR = 2.63; 95% CI 1.57 - 4.39; p < 0.001), which is consistent with other studies [6,8,17]. However, other research has shown that the risk was higher among younger individuals [12,18,19]. Additionally, men had an increased risk of developing severe AEFI compared to women (OR = 1.75; 95% CI 1.05 - 2.90; p = 0.03), which aligns with the South Korean study [8], whereas other studies indicated that the risk is higher in women than in men [12,20,21].

Finally, patients with three or more AEFI also presented a significantly higher risk of developing severe AEFI (OR = 2.08; 95% CI 1.25 - 3.44; p < 0.001) compared to those with one or two AEFI. These results suggest that advanced age, male gender, and a high number of AEFI per patient were associated with severe adverse events.

This study has certain limitations. the absence of certain notification sources, such as the "Yakadaliquah" platform and those directly transmitted to the NPC, to which we did not have access, may have limited the completeness of the data. Additionally, a selection bias in the reporting of AEFI deemed relevant by reporters, as well as missing or incomplete data, may have influenced the representativeness and robustness of the results. Furthermore, because data collection relied on passive surveillance, underreporting, particularly of minor and transient AEFIs, was likely, which may have led to an underestimation of the true frequency of such events.

## Conclusion

The nature, frequency, and variables linked to the severity of AEFI associated with COVID-19 in the Casablanca-Settat region of Morocco were examined in this study. We found that severe AEFI was linked to older age, male gender, and a large number of AEFI per patient. Vigilance is still necessary even though the majority of reported cases were not severe, especially for the communities that have been identified as being at risk. These results further emphasise how crucial it is to have more thorough and reliable surveillance in order to guarantee ongoing vaccine safety monitoring throughout the nation.

### What is known about this topic

- The severity of post-vaccination side effects may vary depending on individual factors such as age, sex, and pre-existing health conditions;
- Most published data come from clinical trials or high-income countries, with limited information from low- and middle-income countries, including Morocco.

### What this study adds

- Older age and male gender increase severity: patients aged ≥65 years (OR = 2.63) and males (OR = 1.75) were more likely to experience severe AEFIs;
- Multiple adverse events per patient raise risk: having ≥3 AEFIs per patient was associated with higher severity (OR = 2.08).
